# Microdialysis and response during regional chemotherapy by isolated limb infusion of melphalan for limb malignancies

**DOI:** 10.1054/bjoc.2001.1902

**Published:** 2001-07

**Authors:** J F Thompson, G A Siebert, Y G Anissimov, B M Smithers, A Doubrovsky, C D Anderson, M S Roberts

**Affiliations:** Department of Surgery, University of Sydney and Sydney Melanoma Unit, Royal Prince Alfred Hospital, Sydney, NSW 2050, Australia; Departments of Medicine, Surgery, University of Queensland, Princess Alexandra Hospital, Brisbane, Qld, 4102, Australia; Department of Dermatology, University of New South Wales, Liverpool Hospital, Liverpool, NSW, 2170, Australia

**Keywords:** regional chemotherapy, microdialysis, isolated limb infusion, melanoma, melphalan

## Abstract

This study sought to use a microdialysis technique to relate clinical and biochemical responses to the time course of melphalan concentrations in the subcutaneous interstitial space and in tumour tissue (melanoma, malignant fibrous histiocytoma, Merkel cell tumour and osteosarcoma) in patients undergoing regional chemotherapy by Isolated Limb Infusion (ILI). 19 patients undergoing ILI for treatment of various limb malignancies were monitored for intra-operative melphalan concentrations in plasma and, using microdialysis, in subcutaneous and tumour tissues. Peak and mean concentrations of melphalan were significantly higher in plasma than in subcutaneous or tumour microdialysate. There was no significant difference between drug peak and mean concentrations in interstitial and tumour tissue, indicating that there was no preferential uptake of melphalan into the tumours. The time course of melphalan in the microdialysate could be described by a pharmacokinetic model which assumed melphalan distributed from the plasma into the interstitial space. The model also accounted for the vascular dispersion of melphalan in the limb. Tumour response in the whole group to treatment was partial response: 53.8% (*n*= 7); complete response: 33.3% (*n*= 5); no response: 6.7% (*n*= 1). There was a significant association between tumour response and melphalan concentrations measured over time in subcutaneous microdialysate (*P*< 0.01). No significant relationship existed between the severity of toxic reactions in the limb or peak plasma creatine phosphokinase levels and peak melphalan microdialysate or plasma concentrations. It is concluded that microdialysis is a technique well suited for measuring concentrations of cytotoxic drug during ILI. The possibility of predicting actual concentrations of cytotoxic drug in the limb during ILI using our model opens an opportunity for improved drug dose calculation. The combination of predicting tissue concentrations and monitoring in microdialysate of subcutaneous tissue could help optimise ILI with regard to post-operative limb morbidity and tumour response. © 2001 Cancer Research Campaign http://www.bjcancer.com


*British Journal of Cancer* (2001) **85**(2), 157–165
© 2001 Cancer Research Campaign
doi: 10.1054/ bjoc.2001.1902, available online at http://www.idealibrary.com on IDEAL®

http://www.bjcancer.com

### Microdialysis and response during regional chemotherapy by isolated limb infusion of melphalan for limb malignancies

**JF Thompson[1], GA Siebert[2], YG Anissimov[2], BM Smithers[3], A Doubrovsky[1], CD Anderson[4] and MS Roberts[2]**

[1]Department of Surgery, University of Sydney and Sydney Melanoma Unit, Royal Prince Alfred Hospital, Sydney, NSW 2050 Australia; Departments of [2]Medicine and [3]Surgery, University of Queensland, Princess Alexandra Hospital, Brisbane, Qld 4102, Australia and [4]Department of Dermatology, University of New South Wales, Liverpool Hospital, Liverpool NSW 2170 Australia

**Summary** This study sought to use a microdialysis technique to relate clinical and biochemical responses to the time course of melphalan concentrations in the subcutaneous interstitial space and in tumour tissue (melanoma, malignant fibrous histiocytoma, Merkel cell tumour and osteosarcoma) in patients undergoing regional chemotherapy by Isolated Limb Infusion (ILI). 19 patients undergoing ILI for treatment of various limb malignancies were monitored for intra-operative melphalan concentrations in plasma and, using microdialysis, in subcutaneous and tumour tissues. Peak and mean concentrations of melphalan were significantly higher in plasma than in subcutaneous or tumour microdialysate. There was no significant difference between drug peak and mean concentrations in interstitial and tumour tissue, indicating that there was no preferential uptake of melphalan into the tumours. The time course of melphalan in the microdialysate could be described by a pharmacokinetic model which assumed melphalan distributed from the plasma into the interstitial space. The model also accounted for the vascular dispersion of melphalan in the limb. Tumour response in the whole group to treatment was partial response: 53.8% ($n$ = 7); complete response: 33.3% ($n$ = 5); no response: 6.7% ($n$ = 1). There was a significant association between tumour response and melphalan concentrations measured over time in subcutaneous microdialysate ($P$ < 0.01). No significant relationship existed between the severity of toxic reactions in the limb or peak plasma creatine phosphokinase levels and peak melphalan microdialysate or plasma concentrations. It is concluded that microdialysis is a technique well suited for measuring concentrations of cytotoxic drug during ILI. The possibility of predicting actual concentrations of cytotoxic drug in the limb during ILI using our model opens an opportunity for improved drug dose calculation. The combination of predicting tissue concentrations and monitoring in microdialysate of subcutaneous tissue could help optimise ILI with regard to post-operative limb morbidity and tumour response. © 2001 Cancer Research Campaign http://www.bjcancer.com

**Keywords**: regional chemotherapy; microdialysis; isolated limb infusion; melanoma; melphalan

Skin cancer is the most common malignancy in Caucasian populations. Melanoma, the most serious form of skin cancer, presents a particularly serious problem as its incidence worldwide continues to rise (Sinha and Benedict, 1996). The treatment of choice for recurrent or in transit melanoma metastases of the limb has been Isolated Limb Perfusion (ILP) since its introduction in 1957 (Creech et al, 1958; Vrouenraets et al, 1996; Duran Garcia et al, 1999). This regional drug administration technique allows concentrations of the cytotoxic agent 15–20 times higher than tolerated systemically to be used (Renard et al, 1995). The drug of choice is the alkylating agent L-phenylalanine mustard (melphalan), administered on its own or in combination with other anti-neoplastic agents, immune-modulators such as TNFα, vasodilators such as papaverine, or anti-neoangiogenic agents. A complete response rate (CR) of 54% and an overall response rate (OR) of 79% have been reported with melphalan used as a single agent (Vrouenraets et al, 1999).

Recently ILP has been replaced in some centres by a simplified method that yields comparable results, Isolated Limb Infusion (Thompson et al, 1994, 1996, 1998). A diagrammatic representation of ILI and sampling by microdialysis is shown in Figure 1. Rapid infusion of the drug into the limb is achieved via percutaneous catheters placed in the axial artery and vein of the limb, followed by recirculation using a syringe and 3-way-tap, and finally washout of residual drug after 20–30 minutes. A heating device is incorporated into the external circuit to maintain limb temperature, but unlike conventional ILP the perfusate is not oxygenated during ILI. The duration of the entire procedure is about one hour and it can potentially be tolerated multiple times even by patients in suboptimal condition. Since 1995 ILI has been successfully performed on a substantial number of patients not only for recurrent limb melanoma, but also for Merkel cell carcinoma and various soft tissue sarcomas in Sydney and Brisbane. We have recently reported pharmacokinetic parameters of melphalan during ILI and presented a mathematical model predicting tissue drug concentrations (Roberts et al, in press).

A problem associated with both ILI and ILP procedures is treatment-induced post operative limb toxicity. As with all forms of chemotherapy, the procedures aim to deliver a maximal drug dose at the highest possible limit of the patient's tolerance. Thus an association of peak drug concentration with limb toxicity would appear likely, however this has yet to be demonstrated (Vrouenraets et al, 1995). As drug concentrations in different tissues such as muscle, skin, fat and tumour vary considerably during ILP and ILI (Scott

*Received 14 December 2000*
*Revised 14 April 2001*
*Accepted 2 May 2001*

*Correspondence to:* MS Roberts

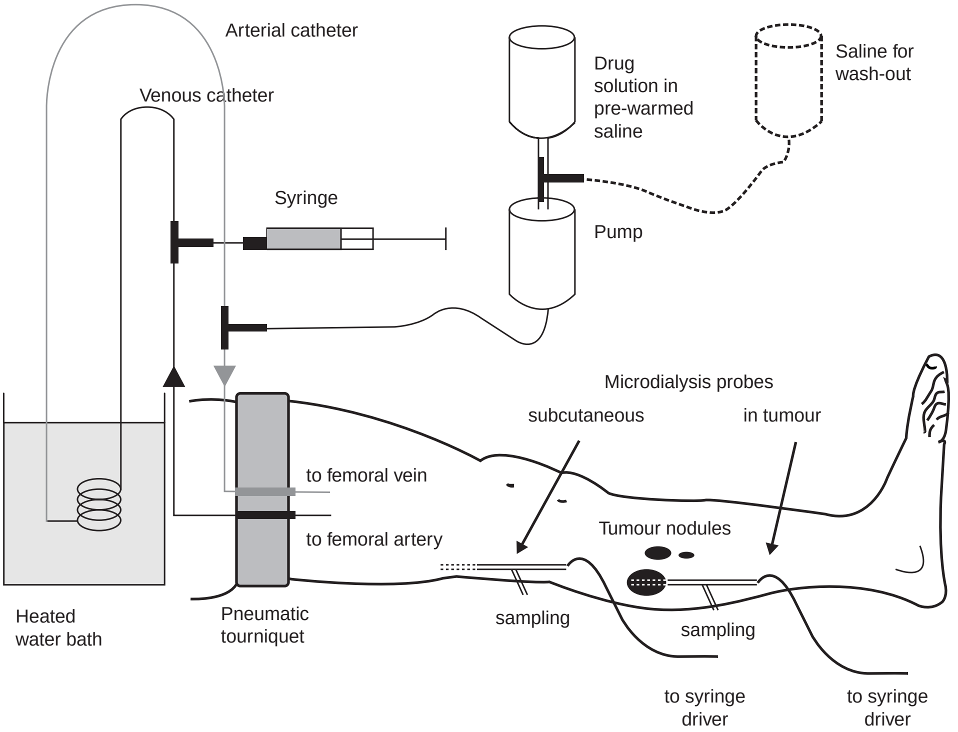

**Figure 1** Diagrammatic representation of ILI and placement of microdialysis probes et al, 1992; Wu et al, 1997a; de Wilt et al, 2000), the respective concentrations cannot readily be derived from plasma concentrations. To obtain data on drug concentrations in normal tissue of patients by performing biopsies is not always practical, and data from tumour biopsies do not reflect drug concentrations in the surrounding tissue, which may, wholly or in part, cause the post-operative toxic reaction. The novel technique of microdialysis allows monitoring of the concentrations of drug in various tissues, and thus permits the relationship between vascular and tissue drug concentrations to be described more exactly (Wu et al, 2000). In this study we have simultaneously monitored plasma concentrations and measured interstitial and tumour concentrations of melphalan by microdialysis in patients undergoing ILI. Microdialysate concentrations were then fitted to a pharmacokinetic model that assumes the microdialysis probe is in the interstitium, with melphalan distribution occurring from and to the plasma. Potential relationships between tumour responses, melphalan toxicity and melphalan pharmacokinetics were then studied.

### PATIENTS AND METHODS

Patients in the study were treated by ILI between August 1998 and September 1999 for multiple cutaneous and/or subcutaneous recurrences of melanoma on a lower limb ($n$ = 14) or upper limb ($n$ = 1), malignant fibrous histiocytoma (lower limb, $n$ = 2), osteosarcoma (lower limb, $n$ = 1) and Merkel cell tumour (lower limb, $n$ = 1) using melphalan and actinomycin D. The study was approved by the institutional Ethics Committee and informed consent for the use of the microdialysis probes during ILI was obtained from all patients treated. Patient demographic data are shown in Table 1.

The protocol for ILI was as described previously (Thompson et al, 1994, 1998). Briefly, venous and arterial catheters were inserted percutaneously before the procedure via the contra-lateral groin and their tips positioned in the affected limb. A pneumatic tourniquet was placed at an appropriate level around the limb of the anaesthetized and heparinized patient, and if the foot or hand was not involved with tumour, it was excluded from infusion by application of an Esmarch rubber bandage. After inflation of the tourniquet, the melphalan solution was rapidly infused into the affected limb, recirculated by withdrawal from the venous catheter and reinjection into the arterial catheter with a syringe using a 3-way tap. After approximately 30 minutes residual drug was flushed from the limb with Hartmann's solution and discarded. Samples were taken from the venous catheter at appropriate intervals for estimation of drug concentrations, blood gases and pH.

The post-operative regional toxicity was graded according to the system proposed by Wieberdink et al (Wieberdink et al, 1982); I: no visible reaction; II: slight erythema and/or oedema; III: considerable erythema and/or oedema with some blistering, with or without slightly disturbed mobility; IV: extensive dermolysis and/or obvious damage to deep tissues, causing definite functional disturbances, or threatened or actual compartment compression syndromes.

Tumour response to chemotherapy was graded and assessed according to standard WHO criteria: CR (complete response): complete disappearance of measurable tumour for at least 4 weeks; PR (partial response): reduction in tumour size of 50% or more; NR (no response): increase of tumour size by more than 25%; stable disease (SD): change of tumour size < 25% to –50% as compared to pre-therapy conditions.

### Microdialysis

The principles of sampling by microdialysis have been described previously (Ungerstedt, 1991; Blochl-Daum et al, 1996; Wu et al, 2000). A diagrammatic representation of the procedure is shown in Figure 2. In brief, the technique is based on the diffusion of

 © 2001 Cancer Research Campaign

**Table 1** Patient demographic data and data pertinent to ILI

| No | Age (years) | Weight (kg) | $V_{inf}$ (ml) | Dose (mg) | Dose (µg ml$^{-1}$) | $T_{on}$ (min) | $T_{inf}$ (min) | $V_{rec}$ (ml) | MD flow (µl min$^{-1}$) | Peak CPK/day (IU l$^{-1}$) | Reaction (Wieberdink) | Tumour response (WHO) | Diagnosis |
|---|---|---|---|---|---|---|---|---|---|---|---|---|---|
| 1 | 56 | 53.4 | 4500 | 35 | 7.8 | 58 | 8 | 1875 | 2.1 | 3740/5 | II | PR | Melanoma |
| 2 | 56 | 69.4 | 3500 | 30 | 8.6 | 63 | 2 | 1450 | 3.5 | 170/2 | II | PR | Melanoma |
| 3 | 74 | 59.5 | 5000 | 37.5 | 7.5 | 60 | 3 | 1525 | 3.5 | 1584/5 | III | CR | Melanoma |
| 4 | 67 | 82.0 | 2300 | 20 | 8.7 | 66 | 2 | 1900 | 3.5 | 331/5 | III | CR | Melanoma |
| 5 | 40 | 87.0 | 7000 | 60 | 8.6 | 74 | 5 | 1600 | 3.5 | 374/4 | II | PR | Melanoma |
| 6 | 76 | 95.6 | 5500 | 45 | 8.2 | 59 | 5 | 2025 | 3.5 | 1279/4 | III | PR | Melanoma |
| 7 | 70 | 58.0 | 3500 | 40 | 7.9 | 65 | 6 | 1250 | 2.0. | 931/1 | II | ** | Melanoma |
| 8 | 70 | 78.0 | 1300 | 45 | 15.4 | 64 | 7 | 1025 | 5.0 | 128/4 | II | CR | Melanoma |
| 9 | 64 | 85.4 | 4400 | 35 | 8.0 | 63 | 3 | 1200 | 5.0 | 184/5 | II | PR | Melanoma |
| 10 | 72 | 98.0 | 12000 | 27.5 | 4.1 | 58 | 5 | 1625 | 5.0 | 1384/5 | II | PR | Melanoma |
| 11 | 43 | 58.0 | 8000 | 20 | 6.3 | 68 | 4 | 3150 | 5.0 | 25/6 | III | * | Melanoma |
| 12 | 33 | 82.0 | 9000 | 35 | 5.6 | 53 | 4 | 1375 | 5.0 | 9464/5 | III | CR | Melanoma |
| 13 | 69 | 85.0 | 7700 | 50 | 6.5 | 67 | 4 | 1300 | 5.0 | 1159/3 | II | CR | Melanoma |
| 14 | 70 | 96.0 | 9000 | 50 | 5.6 | 70 | 5 | 1600 | 5.0 | 1021/4 | III | PR | Melanoma |
| 15 | 64 | 90.0 | 8500 | 50 | 5.9 | 70 | 5 | 1375 | 5.0 | 3466/7 | III | NR | Melanoma |
| 16 | 71 | 74.0 | 4500 | 50 | 8.9 | 65 | 5 | 1800 | 3.5 | 4127/4 | IV | PR | Malignant fibrous histiocytoma |
| 17 | 78 | 53.0 | 4500 | 50 | 10.0 | 61 | 3 | 1350 | 3.5 | 2976/5 | III | PR | Malignant fibrous histiocytoma |
| 18 | 76 | 73.0 | 6500 | 50 | 7.7 | 37 | 5 | 1225 | 5.0 | 3673/6 | III | CR | Merkel cell tumour |
| 19 | 45 | 69.0 | 4500 | 50 | 7.8 | 66 | 6 | 1525 | 3.5 | 198/7 | II | * | Osteosarcoma |
| Mean | 62.8 | 76.1 | 5850 | 41.1 | 7.9 | 62.5 | 4.58 | 1588 | | 1906 | | | |
| SD | 13.5 | 14.7 | 2680 | 11.2 | 2.3 | 7.95 | 1.57 | 461 | | 2305 | | | |

No: patient number; Age: age at time of ILI (years); Weight: body weight at time of ILI (years); $V_{inf}$: Volume of limb infused (ml); Dose (mg): total dose of melphalan given; Dose (µg ml$^{-1}$) melphalan concentration per ml of infused limb; $T_{on}$: period between closing and re-opening of tourniquet; $T_{inf}$: time of rapid drug infusion; $V_{rec}$: volume of manual recirculation of drug (ml); MD flow: flow rate setting of microdialysis probe (µl min$^{-1}$); Probe: type of microdialysis probe used; Peak CPK: peak concentration of CPK (IU l$^{-1}$); Reaction: regional post-operative toxicity, graded as by Wieberdink; Tumour response: graded as by WHO. CR (complete response) complete disappearance of measurable tumour for at least 4 weeks; PR (partial response) reduction in tumour size of 50% or more; NR (no response) increase of tumour size by more than 25%; * no assessment possible – patient moved overseas; ** no assessment possible – patient died of systemic disease; Diagnosis: tumour type.

© 2001 Cancer Research Campaign

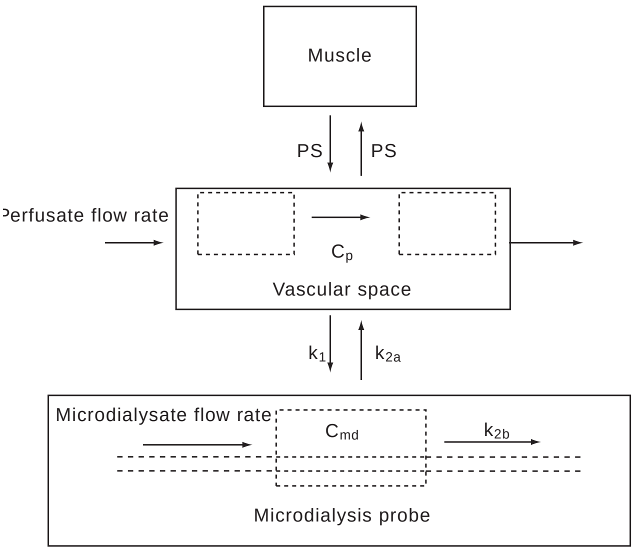

**Figure 2** Diagrammatic representation of the pharmacokinetic model used to describe the distribution of melphalan from plasma into the environment of the microdialysis probe. PS: permeability surface area product from perfusate into muscle; $C_p$: drug concentration in perfusate; $C_{md}$: drug concentration in microdialysate; $k_1$: exchange coefficient between vascular and microdialysis spaces; $k_{2a}$: exchange coefficient between microdialysis and vascular spaces; $k_{2b}$: rate constant for melphalan removal from microdialysis space by microdialysate flow analytes from the interstitial compartment through the semipermeable membrane (cutoff 20 kDalton) of a perfused probe placed in the tissue. Samples of interstitial fluid obtained by microdialysis were collected by inserting a sterile, commercially available microdialysis catheter with a needle subcutaneously into normal or into tumour tissue of the affected limb (CMA 60 or CMA 70, CMA, Solna, Sweden). Perfusion fluid (CMA) was pumped through the catheter with a microdialysis pump (CMA 106 or 107, CMA or MS26 Daily Rate Syringe Driver, SIMS Graseby, Watford, UK). The syringe driver was set at a constant flow rate during the procedure (2, 2.1, 3.47 or 5 µl min$^{-1}$ perfusion fluid). Validity of results and recovery of melphalan under these conditions have been discussed previously (Ungerstedt, 1991; Blochl-Daum et al, 1996; Wu et al, 2000).

### Melphalan dosage

The mean dose of cytotoxic drug (melphalan) was 7.9 ± 2.3 µg ml$^{-1}$ volume of infused limb, with actinomycin D at a concentration $10^{-2}$ that of melphalan. The volume of perfused limbs and of the hand or foot excluded were determined using a simple water displacement technique. Data pertinent to patients and ILIs are summarized in Table 1

### Determination of melphalan concentrations

The plasma samples taken during ILI and the microdialysate specimens were analysed using a HPLC assay developed in our laboratory. The assay, inter and intra assay quality, analytical recovery, linearity, precision and sensitivity have been described previously (Wu et al, 1995). Briefly, 100 µl samples were precipitated with 200 µl methanol containing dansyl-arginine (Sigma) as an internal standard. Fluorescent detection was used with excitation and emission for melphalan of 265 nm and 360 nm, and 265 and 575 nm for the internal standard. The mobile phase consisted of methanol-water-glacial acetic acid (25:75:2 v/v), pH 2.7, with 1-octanosulphonic acid (50 mg 100 ml$^{-1}$), a flow rate of 2 ml min$^{-1}$ and an injection volume of 20 µl onto a phenyl column.

### Model development

The modelling of the melphalan distribution in the plasma of the limb during ILI has been described in detail elsewhere (Roberts et al, in press). This model was extended in this analysis to include the microdialysis site. Figure 2 shows a schematic representation of the model. In brief, the concentration in the microdialysate ($C_{md}$) over time is described by the equation.

$$\frac{d}{dt} C_{md} = k_l C_b - k_2 C_{md}$$

Where $C_b$ is the average perfusate concentration in the two vascular compartments (Figure 2), where $k_1$ is the rate constant for transfer from the perfusate to the microdialysate and $k_2$ is the rate constant for disappearance from the microdialysate (and is the sum of efflux with microdialysate flow ($k_{2b}$) plus transfer from microdialysate to perfusate (Figure 2, $k_{2a}$) ). Other more complex expressions, which recognized exchange into adjunct interstitial space were considered. These are not presented here as they were found not to improve the modelling.

### Data analysis

The non-linear regression program Scientist™ (Micromath Scientific Software, Salt Lake City) was used to numerically integrate 4 differential equations to fit the melphalan data obtained from the infusate and wash-out using weighted ($1/Y_{obs}$) least squares with Hartley modification of the Gauss–Newton algorithm. The final model fitting was deemed acceptable on the basis of the regression goodness-of-fit criteria (MSC, model selection criteria, Scientist) that represented the modified Akaike information criteria (Landaw and DiStefano, 1984), a lack of systemic deviation in the residuals, and the percentage of data accounted for by the regression ($R^2 > 0.99$).

AUCs from zero time to the last sampling time were calculated by trapezoidal rule using Prism 3.0 software.

### RESULTS

A typical representation of the melphalan concentration profile during ILI in plasma and subcutaneous microdialysate and in tumour tissue is shown in Figure 4. Samples were usually collected over 5 min intervals during infusion and recirculation of the drug (plasma and microdialysis data) and over 15 min after time point 75 min (microdialysis). The drug concentrations which were monitored followed a similar pattern in the 3 tissues, with a time delay of 5 min for peak concentrations of subcutaneous and tumour tissues when compared to plasma (Figure 4).

The results of plasma and microdialysis measurements of the patients made during ILI are shown in Table 2. One patient was excluded from calculation of mean tumour melphalan concentration due to an aberrant value.

Peak concentrations and AUCs of melphalan were higher in plasma (24.8 ± 6.1 µg ml$^{-1}$ and 516 ± 121 µg min$^{-1}$ ml$^{-1}$) than in microdialysis samples from subcutaneous tissue (10.3 ± 5.1 µg

© 2001 Cancer Research Campaign

**Table 2** Peak concentrations of melphalan in perfusate, subcutaneous and tumour dialysate and pharmacokinetic parameters

| | Peak concentrations | | | | | Pharmacokinetic parameters | | | | |
|---|---|---|---|---|---|---|---|---|---|---|
| | Perfusate | Microdialysis | | | | | | | | |
| | $C_p$ | $C_{md\ s\ c}$ | | $C_{md\ tum}$ | | $AUC_p$ | $AUC_{md}$ | $AUC_{tum}$ | k1/k2 | $PS_t$ |
| | (μg ml$^{-1}$) | (μg ml$^{-1}$) | % (pl) | (μg ml$^{-1}$) | % (pl) | (μl min$^{-1}$ ml$^{-1}$) | (μl min$^{-1}$ ml$^{-1}$) | (μl min$^{-1}$ ml$^{-1}$) | | (ml min$^{-1}$ 100 g$^{-1}$) |
| 1 | 22.3 | 5.7 | 25.6 | | | 488 | 137 | | 0.32 | 4.6 |
| 2 | 27.2 | 5.8 | 21.3 | | | 630 | 418 | | 0.28 | 0.3 |
| 3 | 31.3 | 15.1 | 48.2 | | | 646 | 420 | | 0.39 | 1.0 |
| 4 | 11.8 | 15.3 | 130 | | | 254 | 429 | | 0.69 | 1.0 |
| 5 | 13.8 | 4.6 | 33.3 | | | 349 | 113 | | 0.10 | 5.5 |
| 7 | 33.2 | 13.9 | 41.9 | | | 801 | 300 | | 0.15 | 0.6 |
| 8 | 33.5 | 9.6 | 28.7 | 13.7 | 40.9 | 666 | 497 | 439 | 0.48 | 1.1 |
| 9 | 23.1 | 7.5 | 32.5 | | | 480 | 345 | | 0.54 | 1.3 |
| 10 | 24.1 | 8.4 | 34.9 | | | 503 | 177 | | 0.20 | 0.8 |
| 11 | 17.8 | 14.6 | 82.0 | 14.0 | 78.7 | 441 | 211 | * | 0.26 | 0.6 |
| 12 | 28.3 | 21 | 74.2 | 12.5 | 44.2 | 517 | 18 | 412 | | 1.0 |
| 13 | 28.2 | 12.8 | 45.4 | | | 528 | 423 | | 0.47 | 1.9 |
| 14 | 21.3 | 17.1 | 80.3 | *48.6* | *228* | 424 | 204 | *1071* | 0.38 | 4.0 |
| 15 | 30.2 | 10.7 | 35.4 | 11.4 | 37.7 | 523 | 376 | 231 | 0.38 | 1.8 |
| 16 | 23.6 | 9.5 | 40.3 | | | 511 | 691 | | 0.65 | 2.0 |
| 17 | 22.3 | 5.2 | 23.3 | 2.0 | 9.0 | 522 | 312 | 78 | 0.27 | 1.6 |
| 18 | 26.5 | 7.1 | 26.8 | 2 | 7.5 | 531 | 307 | 74 | 0.31 | 1.6 |
| 19 | 28.4 | 2.2 | 7.7 | 1.2 | 4.2 | 478 | 82 | 9 | 0.07 | 2.4 |
| Mean | 24.8 | 10.3 | 45.1 | 8.1 | 31.7 | 516 | 303 | 207 | 0.35 | 1.8 |
| S D | 6.1 | 5.1 | 29.3 | 6.0 | 26.9 | 121 | 168 | 184 | 0.18 | 1.5 |
| Median | 25.3 | 9.6 | 35.2 | 11.4 | 37.7 | 514 | 310 | 155 | 0.32 | 1.5 |

$C_p$: melphalan concentration in perfusate (μg/ml); % (p): percentage of melphalan perfusate concentration; $C_{md\ sc}$: melphalan concentration in subcutaneous microdialysate (μg ml$^{-1}$); $C_{md\ tum}$: melphalan concentration in tumour microdialysate (μg ml$^{-1}$); AUC: area under curve; $AUC_p$: area under curve melphalan area under curve in perfusateversus time; $AUC_{md}$: melphalan area under curve in microdialysate versus time; $AUC_{tum}$: melphalan area under curve in tumour versus time; $k_1/k_2$ and $PS_t$ are defined in Fig. 2; *insufficient data points; *values in italics* were excluded from statistics.

© 2001 Cancer Research Campaign

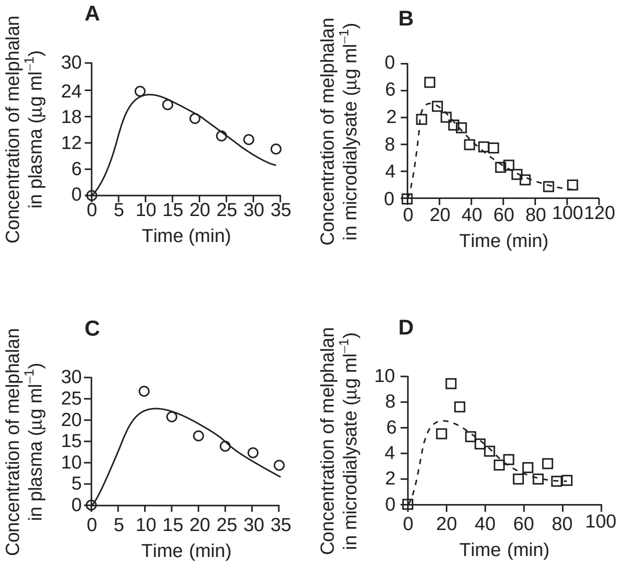

**Figure 3** Melphalan concentrations measured in two typical patients during Isolated Limb Infusion in plasma (A, C, open symbols) and data predicted by the model (A, C solid line) and in microdialysate (B, D, dotted line); open symbols: drug concentrations measured in microdialysate

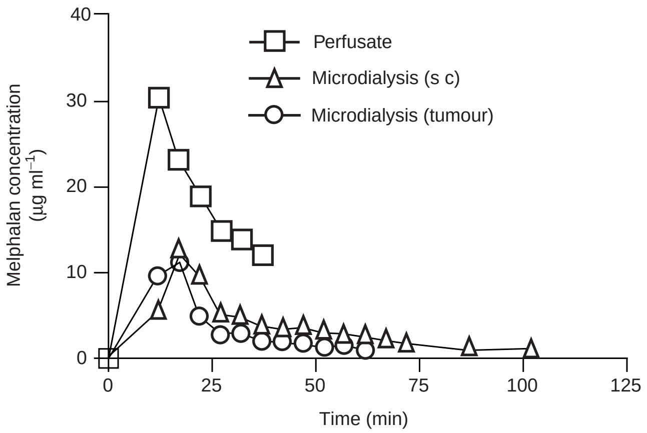

**Figure 4** Typical melphalan concentration profiles in perfusate, in outflow effluents from microdialysis probes in subcutaneous tissue and in melanomas during ILI

$ml^{-1}$ and 303 ± 168 µg $min^{-1}$ $ml^{-1}$) or tumour microdialysis samples (8.1 ± 6.0 µg $ml^{-1}$ and 207 ± 184 µg $min^{-1}$ $ml^{-1}$, mean ± standard deviation, Table 2). A higher variability for drug peak concentrations was observed in tumour tissue (see patient 14, Table 2, data excluded), and the concentration differences between subcutaneous and tumour microdialysate were not significant ($P > 0.05$, Student's *t*-test). There were significant differences, however, between the peak plasma concentrations and the concentrations in subcutaneous and tumour microdialysate ($P < 0.001$). The same differences were found for comparison of the AUCs (Table 2).

A comparison between predictions of melphalan concentrations in plasma and interstitial space according to the model with actual concentrations in two representative studies are shown in Figure 3.

Peak and median concentrations of melphalan measured during ILI, melphalan AUCs and pharmacokinetic parameters are presented in Table 2. The permeability surface area product (PS) values for transfer of melphalan from perfusate into muscle showed some degree of variability (1.8 ± 1.5 ml $min^{-1}$ 100 $g^{-1}$ but were comparable with earlier results; Roberts et al, in press). The relationships between measured drug concentrations in plasma and microdialysate, calculated rations ($k_1/k_2$) of the influx rate constant into microdialysate from perfusate ($k_1$) to efflux from microdialysate ($k_2$), grade of post-operative toxicity and peak CPK values are shown in Table 2 and Figure 5. No significant relationship between the grade of limb toxicity and melphalan concentration in plasma or microdialysate could be found (Figure 5 A, C, $P > 0.05$). There was no significant difference ($P > 0.05$) to prove that the higher ratios of rate constants for influx into a microdialysate from perfusate relative to efflux ($k_1/k_2$ value > 0.35) was associated with elevated limb toxicity, however a larger number of patients would be needed to decide the question (Figure 5 E). No relationship was found to exist between peak CPK concentrations and melphalan peak concentrations in plasma or microdialysate and between CPK concentrations and the influx from perfusate into microdialysate to efflux ratio ($k_1/k_2$. Figure 5 B, D, F, $P > 0.05$).

Analysis of the relationship between tumour response (comparison of poor and good responders, PR and NR, and CR, respectively) to treatment and melphalan concentrations reached during ILI showed no significant difference with regards to the drug peak concentrations or AUC. The mean concentrations of subcutaneous microdialysate, however, were significantly higher ($P < 0.01$) in patients that showed complete response (5.34 ± 0.44 µg $ml^{-1}$) compared to those that showed only partial or no response (3.72 ± 0.33 µg $ml^{-1}$).

### DISCUSSION

In this study, measurements of melphalan concentrations were made by microdialysis in a group of patients undergoing ILI for various limb tumours, allowing drug concentration/time profiles to be constructed, and drug concentrations achieved in different tissues during ILI to be compared (Figure 4).

Microdialysis has been successfully employed before for measuring concentrations of various cytotoxic drugs in the interstitial space of different tissues and tumours in vivo. The transfer of cytotoxic drugs from plasma to interstitial space and finally across the tumour endothelium into the intracellular tumour space is considered a critical step in tumour resistance to chemotherapy and microdialysis therefore is an important tool in assessing interstitial tissue pharmacokinetics and plasma-to-tissue transfer rates of cytotoxic drugs in human patients. Clinically, microdialysis has been used to measure 5-fluorouracil concentrations in breast cancer patients (Muller et al, 1997; Mader et al, 1998), where high interstitial tumour concentrations of the drug were reported to be associated with an improved tumour response, whereas plasma and subcutaneous drug levels showed no correlation to tumour response (Muller et al, 1997). Contrasting results have been reported for breast cancer patients and a patient treated for malignant fibrous histiocytoma (MFH) with methotrexate: analysis of microdialysates of the breast cancer patients showed no correlation between plasma, interstitial and tumour drug concentrations (Muller et al, 1998), whereas a close correlation was found between tumour and systemic levels for both methotrexate and its major extracellular metabolite 7-hydroxymethotrexate in microdialysate of the MFH patient (Ekstrom et al, 1997). Analysis of intratumoral and subcutaneous concentrations of carboplatin in

 *© 2001 Cancer Research Campaign*

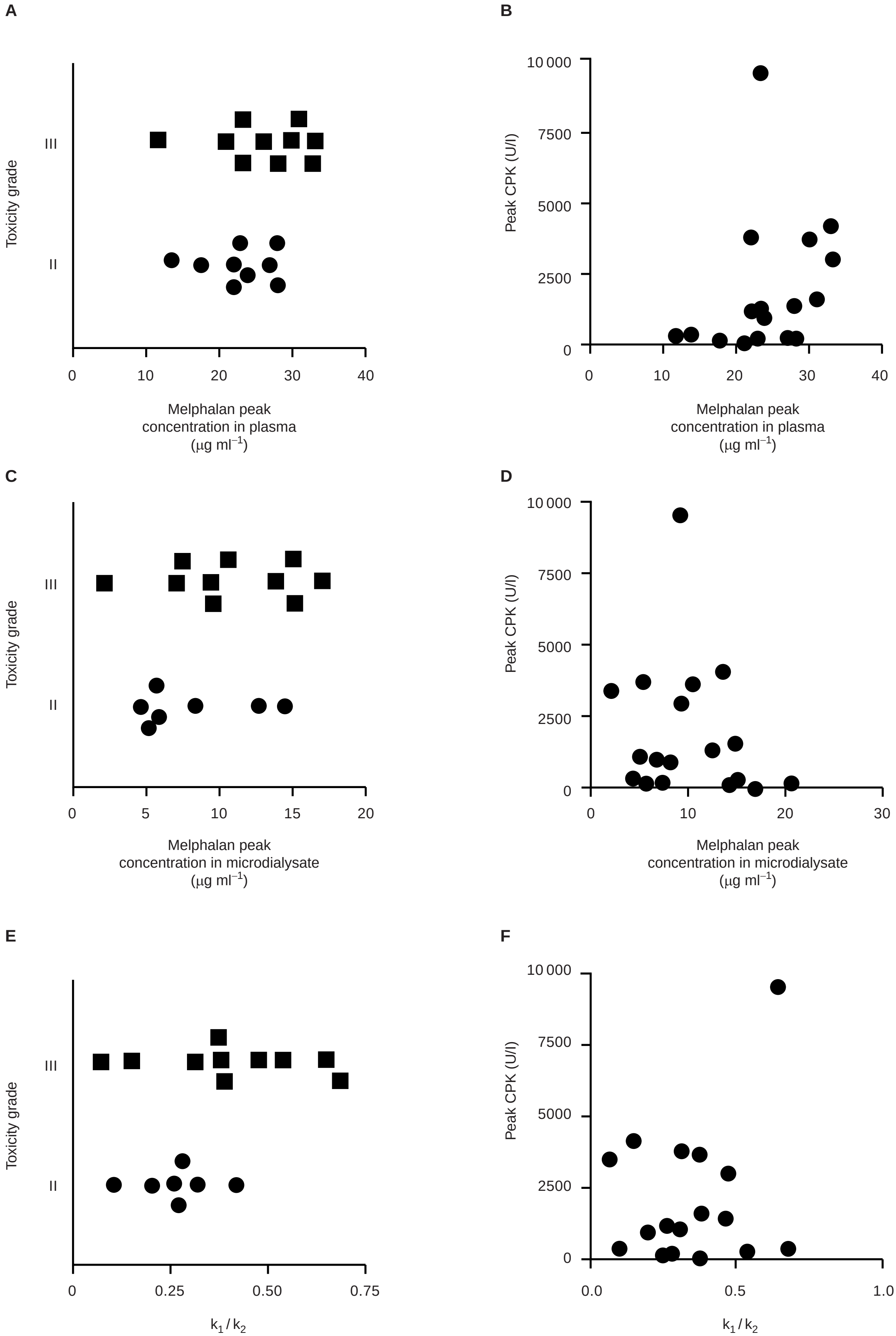

**Figure 5** Relationships between degree of toxicity and (**A**) melphalan peak concentration in plasma, (**C**) melphalan peak concentration in microdialysate and (**E**) influx and efflux rate constants ratio. Relationships between CPK peak concentration and (**B**) melphalan peak concentration in plasma, (**D**) melphalan peak concentration in microdialysate and (**F**) influx and efflux rate constants ratio

*© 2001 Cancer Research Campaign*

microdialysate of patients treated by systemic administration of the drug for melanoma did not show any difference between subcutaneous and tumour tissue (Blochl-Daum et al, 1996).

Technical differences between ILI and ILP such as lower circulation flow rates, more normothermic conditions and a comparatively more hypoxic and acidotic perfusate during ILI (Thompson et al, 1996) could be responsible.

In our study, the difference between peak drug concentrations or AUCs in subcutaneous and tumour microdialysate was not statistically significant. After exclusion of the unrealistic drug concentration in tumour dialysate from patient 14 the peak concentration mean was 8.1 ± 6.0 μg $ml^{-1}$ and there was no difference between drug concentrations in the microdialysate in subcutaneous and tumour tissues.

Analysis of drug concentrations over time in both tissues of 6 patients again showed no differences between tumour and skin concentrations, which is in agreement with the previous results from an animal model (Wu et al, 1997a). It remains to be clarified, however, whether a contradictory report (Klaase et al, 1994) reflects differences in protocol or, as is more likely, variations in individual tumour physiology with respect to factors such as their origin, site, size, and particularly their vascularisation, local blood flow and degree of necrosis and development of haematoma during the implantation of microdialysis probe. Also, measurements taken from tumour tissue by microdialysis must generally be evaluated with great care (small tumour nodules may for example not cover the entire length of the microdialysis probe).

No association between the various pharmacokinetic parameters tested and the treatment-induced post-operative toxicity could be established. No obvious relation between peak drug concentrations and limb toxicity has previously been recorded and it was absent in this study for plasma, subcutaneous and tumour tissue. Drug distribution parameters such as PS values or exchange rates are theoretical candidates for involvement in the toxic limb reaction. A weak, non-significant relation between the ratio for the exchange rates $k_1/k_2$ and the degree of limb toxicity was observed, however, and a study of a greater number of patients is warranted to either prove the relationship or to exclude it. An association of 'late' CPK peaks analogous to the association between the 'late peak CPK' with elevated post-operative limb toxicity (Lai et al, 1993) could not be established in our sample (data not shown); however, this result too could possibly be attributable to the modest sample size.

In this study there was a significant correlation between the mean melphalan concentration in subcutaneous microdialysate over time and the tumour response. There are some possible reasons why this relationship could be demonstrated for drug concentrations in healthy tissue but not for tumour microdialysate. The anatomical subcutaneous environment in which the microdialysis probe is placed is possibly more constant than that of the probes placed in tumour nodules. The varying degree of tumour necrosis and resulting changes in contact area of the probe with the interstitial space could well be responsible for artificially low results. Differences in micro- and macro-tumour vasculature are also potential sources of variation. The higher variability of data values measured in tumour tissue compared with data derived from subcutaneous tissue or plasma could be indicative of problems associated with these factors. More data will be needed, however, to confirm the association of subcutaneous drug concentration with tumour response.

All patients received actinomycin D at a concentration one hundredth that of melphalan during ILI. We believe that the contribution of actinomycin D at this concentration to either activity or toxicity of the treatment was minimal. Based on our personal experience comparing outcomes in a large prophylactic ILP trial carried out by the WHO/EORTC with no actinomycin D; (Koops et al, 1998) and a large number of therapeutic ILPs with actinomycin D added; (Thompson et al, 1997). However, to allow direct comparability of clinical outcomes following therapeutic ILIs with those following ILPs, we chose not to eliminate the drug from our protocol.

It was one aim of this study to use a pharmacokinetic model to predict drug concentrations in a variety of tissues. The distribution of solutes into tissues can be described by at least 3 models: (i) a quasi-instantaneous solute distribution into tissue caused by rapid diffusion and binding, (ii) a slower distribution of the solute due to rapid binding to cellular structures and/or carrier proteins; (iii) slow binding to intracellular sites after rapid cytosolic distribution of the solute (Weiss et al, 2000). The first model is often used to describe solute distribution as a 2-compartment dispersion or tank-in-series models (Roberts et al, 1988). Models (ii) and (iii) are more complex, taking into account the fact that intracellular drug distribution may not be instantaneous (Weiss et al, 2000). The model chosen to represent the drug distribution during ILI was the less complex 2-compartment tank-in-series model, finding that criteria such as 'goodness of fit' and consistency of parameter estimates (as reflected by coefficients of variation) were within expected limits. The predicted data for subcutaneous drug concentrations varied with respect to initial peak concentrations (Figure 3 B, D).

Introduction of an adjacent interstitial space to the model (Figure 2) changed the predicted values and model selection criteria only marginally. It is possible that these initial peak concentrations could be attributable to measurements of plasma concentrations in the subcutaneous microdialysis probes due to minor, transient haemorrhage from introducing the probes in fully heparinised patients.

It is concluded that microdialysis is a technique well suited for measuring concentrations of cytotoxic drug during the ILI procedure. The results of this study show that the melphalan concentrations in tumour and normal limb tissue during ILI are similar. The possibility of predicting actual concentrations of cytotoxic drug in the limb during ILI using our model opens an opportunity for improved drug dose selection. The combination of predicting tissue concentrations and monitoring achieved mean drug concentrations over time in microdialysate of subcutaneous tissue could help optimising ILI with regards to post-operative limb morbidity and tumour response.

### ACKNOWLEDGEMENTS

The authors wish to acknowledge the financial support of the National Health and Medical Research Council of Australia, the Lions Queensland and Northern New South Wales Medical Research Foundation and the Melanoma Foundation of the University of Sydney. The authors would also like to thank Dr Zhen Yu Wu and Ross Norris for their help in preparing the manuscript.

### REFERENCES

Blochl-Daum B, Muller M, Meisinger V, Eichler HG, Fassolt A and Pehamberger H (1996) Measurement of extracellular fluid carboplatin kinetics in melanoma metastases with microdialysis. *Br J Cancer* **73**: 920–924

© 2001 Cancer Research Campaign

Creech O, Krementz E, Ryan R and Winblad J (1958) Chemotherapy of cancer: Regional perfusion utilizing an extracorporeal circuit. *Ann Surg* **148**: 616–632
de Wilt JH, ten Hagen TL, de Boeck G, van Tiel ST, de Bruijn EA and Eggermont AM (2000) Tumour necrosis factor alpha increases melphalan concentration in tumour tissue after isolated limb perfusion. *Br J Cancer* **82**: 1000–1003
Duran Garcia, E, Santolaya R and Requena T (1999) Treatment of malignant melanoma. *Ann Pharmacother* **33**: 730–738
Ekstrom PO, Andersen A, Saeter G, Giercksky KE and Slordal L (1997) Continuous intratumoral microdialysis during high-dose methotrexate therapy in a patient with malignant fibrous histiocytoma of the femur: a case report. *Cancer Chemother Pharmacol* **39**: 267–272
Klaase JM, Kroon BB, Beijnen JH, van Slooten GW and van Dongen JA (1994) Melphalan tissue concentrations in patients treated with regional isolated perfusion for melanoma of the lower limb. *Br J Cancer* **70**: 151–153
Koops HS, Vaglini M, Suciu S, Kroon BB, Thompson JF, Gohl J, Eggermont AM, Di Fillippo F, Krementz ET, Ruiter D and Lejeune FJ (1998) Prophylactic isolated limb perfusion for localized, high-risk limb melanoma: results of a multicenter randomized phase III trial. European Organization for Research and Treatment of Cancer Malignant Melanoma Cooperative Group Protocol 18832, the World Health Organization Melanoma Program Trial 15, and the North American Perfusion Group Southwest Oncology Group-8593. *J Clin Oncol* **16**: 2906–2912
Lai D, Ingvar C and Thompson J (1993) The value of monitoring serum creatine phosphokinase following hyperthermic isolated limb perfusion for melanoma. *Reg Cancer Treat* **1**: 36–39
Landaw EM and DiStefano JJd (1984) Multiexponential, multicompartmental, and noncompartmental modeling. II. Data analysis and statistical considerations. *Am J Physiol* **246**: R665–R677
Mader RM, Brunner M, Rizovski B, Mensik C, Steger GG, Eichler HG and Muller M (1998) Analysis of microdialysates from cancer patients by capillary electrophoresis. *Electrophoresis* **19**: 2981–2985
Muller M, Mader RM, Steiner B, Steger GG, Jansen B, Gnant M, Helbich T, Jakesz R, Eichler HG and Blochl-Daum B (1997) 5-fluorouracil kinetics in the interstitial tumor space: clinical response in breast cancer patients. *Cancer Res* **57**: 2598–2601
Muller M, Brunner M, Schmid R, Mader RM, Bockenheimer J, Steger GG, Steiner B, Eichler HG and Blochl-Daum B (1998) Interstitial methotrexate kinetics in primary breast cancer lesions. *Cancer Res* **58**: 2982–2985
Renard N, Nooijen PT, Schalkwijk L, De Waal RM, Eggermont AM, Lienard D, Kroon BB, Lejeune FJ and Ruiter DJ (1995) VWF release and platelet aggregation in human melanoma after perfusion with TNF alpha. *J Pathol* **176**: 279–287
Roberts MS, Donaldson JD and Rowland M (1988) Models of hepatic elimination: comparison of stochastic models to describe residence time distributions and to predict the influence of drug distribution, enzyme heterogeneity, and systemic recycling on hepatic elimination. *J Pharmacokinet Biopharm* **16**: 41–83
Scott RN, Blackie R, Kerr DJ, Hughes J, Burnside G, MacKie RM, Byrne DS and McKay AJ (1992) Melphalan concentration and distribution in the tissues of tumour-bearing limbs treated by isolated limb perfusion. *Eur J Cancer* **11**: 1811–1813
Sinha T and Benedict R (1996) Relationship between latitude and melanoma incidence: international evidence. *Cancer Lett* **99**: 225–231
Thompson J, Waugh R, Saw R and Kam P (1994) Isolated limb infusion with melphalan for recurrent limb melanoma: a simple alternative to isolated limb perfusion. *Reg Cancer Treat* **7**: 188–192
Thompson JF, Ramzan I, Kam P and Yau D (1996) Pharmacokinetics of melphalan during isolated limb infusion for melanoma. *Regional Cancer Treatment* **9**: 13–16
Thompson JF, Hunt JA, Shannon KF and Kam PC (1997) Frequency and duration of remission after isolated limb perfusion for melanoma. *Arch Surg* **132**: 903–907
Thompson JF, Kam PC, Waugh RC and Harman CR (1998) Isolated limb infusion with cytotoxic agents: a simple alternative to isolated limb perfusion. *Semin Surg Oncol* **14**: 238–247
Ungerstedt U (1991) Microdialysis—principles and applications for studies in animals and man. *J Intern Med* **230**: 365–373
Vrouenraets BC, Kroon BB, Klaase JM, Nieweg OE, van Slooten GW and van Dongen JA (1995) Severe acute regional toxicity after normothermic or ‘mild’ hyperthermic isolated limb perfusion with melphalan for melanoma. *Melanoma Res* **5**: 425–431
Vrouenraets BC, Nieweg OE and Kroon BB (1996) Thirty-five years of isolated limb perfusion for melanoma: indications and results. *Br J Surg* **83**: 1319–1328
Vrouenraets BC, Hart GA, Eggermont AM, Klaase JM, van Geel BN, Nieweg OE and Kroon BB (1999) Relation between limb toxicity and treatment outcomes after isolated limb perfusion for recurrent melanoma. *J Am Coll Surg* **188**: 522–530
Weiss M, Kuhlmann O, Hung D and Roberts M (2000) Cytoplasmic binding and disposition kinetics of diclofenac in the isolated perfused rat liver. *British Journal of Pharmacology*, in press.
Wieberdinck J, Benckhuysen C, Braat RP, van Slooten EA and Olthuis GA (1982) Dosimetry in isolation perfusion of the limbs by assessment of perfused tissue volume and grading of toxic tissue reactions. *Eur J Cancer Clin Oncol* **18**: 905–910
Wu ZY, Thompson MJ, Roberts MS, Addison RS, Cannell GR, Grabs AJ and Smithers BM (1995) High-performance liquid chromatographic assay for the measurement of melphalan and its hydrolysis products in perfusate and plasma and melphalan in tissues from human and rat isolated limb perfusions. *J Chromatogr B Biomed Appl* **673**: 267–279
Wu Z, Roberts MS, Parsons PG and Smithers BM (1997a) Isolated limb perfusion with melphalan for human melanoma xenografts in the hindlimb of nude rats: a surviving animal model. *Melanoma Res* **7**: 19–26
Wu ZY, Smithers BM and Roberts MS (1997b) Melphalan dosing regimens for management of recurrent melanoma by isolated limb perfusion: application of a physiological pharmacokinetic model based on melphalan distribution in the isolated perfused rat hindlimb. *Melanoma Res* **7**: 252–264
Wu ZY, Smithers BM, Anderson C and Roberts MS (2000) Can tissue drug concentrations be monitored by microdialysis during or after isolated limb perfusion for melanoma treatment? *Melanoma Res* **10**: 47–54

*© 2001 Cancer Research Campaign*
